# Outcomes Following Intrasphincteric Injection of Botulinum Toxin for Treatment of Anal Fissures

**DOI:** 10.7759/cureus.53668

**Published:** 2024-02-05

**Authors:** Saania Peeroo, Ashray Rajagopalan, Asiri Arachchi, Samuel Penfold, Blake Roschach, Thang Chien Nguyen, William Teoh

**Affiliations:** 1 Department of Surgery, Monash Health, Melbourne, AUS; 2 Department of Surgery, Monash University, Melbourne, AUS

**Keywords:** faecal incontinence, intrasphincteric, botulinum toxins, anus diseases, fissure in ano

## Abstract

Background

Intrasphincteric injection of botulinum toxin is an alternative treatment for anal fissures, which may present less risk of fecal incontinence than more invasive procedures, such as lateral internal sphincterotomy. The aim is to compare cure and complication rates between these two treatments.

Methods

We conducted a retrospective audit of patients who underwent treatment of anal fissures with intrasphincteric botulinum toxin or lateral internal sphincterotomy from 2016 to 2020 at the Colorectal Surgery Unit of Monash Health, Melbourne, Australia, excluding those who had previously had either procedure.

Results

Fifty-one patients received intrasphincteric botulinum toxin, and 40 patients underwent lateral internal sphincterotomy. Most patients in the botulinum group had a total dose of either 80 (53%; n=27) or 100 units (37%; n=19) and had the dose administered bilaterally at the 3 o’clock and 9 o’clock positions (n=41; 80%). Thirty-one patients in the botulinum group (61%) had complete resolution of symptoms, with a mean time to cure of two months, compared to 36 patients (90%) in the sphincterotomy group with a mean time to cure of 1.5 months. In most cases, postoperative incontinence was transient, although one patient in the botulinum group had persistent incontinence of flatus, and two patients in the sphincterotomy group had persistent fecal incontinence.

Conclusion

Intrasphincteric botulinum injection is an effective, less-invasive alternative to sphincterotomy for the treatment of anal fissures, with incontinence usually temporary when it occurs. Further research is needed to optimize the dose and location of injection and guide future recommendations.

## Introduction

Anal fissures are a common cause of perianal pain and bleeding associated with bowel movements in both men and women [1]. Traumatic tearing of the anoderm from the passage of hard stools has been historically regarded as the main cause; however, the role of increased resting anal tone and reduced anodermal blood flow, with subsequent delayed healing, are now also understood as key to the pathogenesis of chronic anal fissures [2]. Chronic anal fissure is a significant cause of morbidity, with demonstrated improvements in both physical and social functioning, pain, and mental health following treatment [3].

There are a variety of approaches to the management of anal fissures in Australia [4]. Lateral internal sphincterotomy is classically regarded as the gold standard in the treatment of anal fissures, with partial division of the sphincter to reduce anal tone and spasm of the internal anal sphincter. However, disruption of the internal anal sphincter also presents a risk of incontinence of flatus or feces, which has driven interest in alternative, less-invasive treatments. In addition to conservative pharmacological therapies such as topical glyceryl trinitrate ointment, the injection of botulinum toxin A can be used to achieve a temporary chemical sphincterotomy [2,5].

Existing evidence suggests that lateral internal sphincterotomy has superior efficacy in healing and reduced recurrence, although botulinum toxin injection has a lower rate of incontinence [6-9].

However, there remains a lack of consensus on the choice of intrasphincteric botulinum toxin injection versus lateral internal sphincterotomy. Additionally, regimens for the injection of botulinum toxin also vary, with differing dosages and sites of injection between treatment protocols. This study aims to compare cure and complication rates between these two treatments for anal fissures.

This article was previously presented as a meeting abstract at the 2022 ASCRS Annual Scientific Meeting in May 2022 in Florida, USA.

## Materials and methods

We conducted a retrospective audit of all patients who underwent the treatment of chronic anal fissure with intrasphincteric botulinum toxin or lateral internal sphincterotomy from 2016 to 2020 at the Monash Health Colorectal Surgery Unit. Adult patients who received either procedure were eligible for inclusion and identified from operative records. We excluded patients who had previously had either procedure within the preceding 12 months or who were lost to follow-up. As a retrospective quality assurance project, an exemption from formal ethics approval was obtained for this study.

Chronic anal fissure was diagnosed based on the clinical history of anal symptoms of at least eight weeks in duration and upon visualization of the fissure on examination. Most patients had a total botulinum dose of either 80 (53%; n=27) or 100 units (37%; n=19), while the remainder had lower doses. Most (n=41; 80%) had the dose divided into two doses and administered bilaterally at the 3 o’clock and 9 o’clock positions; three (6%) had the dose administered at 3 o’clock, 9 o’clock as well as locally to the fissure site, while five (10%) had the dose divided and administered into four quadrants at the 12 o’clock, 3 o’clock, 6 o’clock, and 9 o’clock positions. The botulinum toxin dosages and sites of administration are provided in Table 1.

**Table 1:** Dosage and sites of botulinum injection

Dosage and sites of botulinum injection	Botulinum n(%)
Total dose of botulinum (IU)	
40	1 (2%)
60	3 (6%)
70	1 (2%)
80	27 (53%)
100	19 (37%)
Site of administration	
Bilaterally	41 (80%)
Bilaterally and locally at the site of the fissure	3 (6%)
Four quadrants	5 (10%)
Locally at the site of the fissure	1 (2%)
Unilaterally	1 (2%)

Data were collected from the medical record and from a single follow-up telephone survey conducted in July or August 2021. Baseline patient characteristics were compared between the two groups, including demographic data (age and sex) and position of the fissure, duration of symptoms, and previous treatments. Outcomes of interest were the proportion of patients with complete resolution of symptoms, time to cure and rate of reintervention because of recurrence of symptoms, and the occurrence and duration of any postoperative fecal incontinence. For patients receiving botulinum toxin injection, we also identified the total dose and the sites of injection. Subgroup analysis was also performed to compare outcomes for male and female patients in each group.

Statistical analysis was performed with Statistical Product and Service Solutions (SPSS, version 27; IBM SPSS Statistics for Windows, Armonk, NY). We compared outcomes between groups using Fisher’s exact test for categorical variables and using the independent samples t-test for continuous variables. A two-tailed p<0.05 was considered statistically significant.

## Results

Fifty-one patients received intrasphincteric botulinum toxin, and 40 patients received lateral internal sphincterotomy during the study period. Most patients receiving botulinum were female (78%; n=40), while most sphincterotomy patients were male (73%; n=29). Seven patients had a fissure attributable to vaginal delivery in the botulinum group, and one patient in the sphincterotomy group. The baseline patient characteristics are provided in Table 2. Median follow-up was 36 months (range 7-65 months).

**Table 2:** Baseline characteristics

Baseline characteristics	Botulinum n(%) or mean±standard deviation	Sphincterotomy n(%) or mean±standard deviation
Age (years)	41.5±14.3	43.9±13.1
Sex (male:female)	11:40 (22%:78%)	29:11 (73%:28%)
Position of fissure		
Anterior	18 (35%)	3 (8%)
Posterior	30 (59%)	34 (85%)
Multiple	3 (6%)	3 (8%)
Parous female	28 (55%)	8 (20%)
Fissure attributable to vaginal delivery	7 (14%)	1 (3%)
Previous rectal or anal surgery	11 (22%)	4 (10%)

Thirty-one patients in the botulinum group (61%) had complete resolution of symptoms, with a mean time to cure of two months. This was compared to 36 patients (90%) in the sphincterotomy group with a mean time to cure of 1.5 months. This difference in cure rate was statistically significant (p=0.002). Eleven patients in the botulinum group (22%) and four patients in the sphincterotomy group (10%) reported incontinence postoperatively. In most cases, this was transient, although one patient in the botulinum group had persistent incontinence of flatus, and two patients in the sphincterotomy group had persistent fecal incontinence. There was no statistically significant difference between groups concerning postoperative complications. The postoperative outcomes are provided in Table 3.

**Table 3:** Postoperative outcomes †1 patient in the botulinum group had persistent incontinence of flatus, and two patients in the sphincterotomy group had persistent fecal incontinence.

Postoperative outcomes	Botulinum n(%) or mean±standard deviation	Sphincterotomy n(%) or mean±standard deviation
Healing of fissure	31 (61%)	36 (90%)
Time to healing (months)	2.0±1.9	1.5±1.5
Occurrence of incontinence	11 (22%)	4 (10%)
· Feces only	7 (14%)	1 (2%)
· Flatus only	2 (4%)	0 (0%)
· Faeces and flatus	2 (4%)	3 (7%)
Time to resolution of incontinence (months)†	2.9±2.4	1.0±0.0
Requiring botulinum reinjection	7 (14%)	
· One reinjection	6 (12%)	
· Two or more reinjections	1 (2%)	
Time to reinjection (months)	6.7±6.2	
Requiring further surgery	10 (20%)	5 (13%)
Time to further surgery (months)	7.7±7.6	3.7±2.8

Subgroup analysis by sex did not demonstrate a statistically significant difference in the rate of healing, incontinence, need for reinjection, or recurrence requiring further surgery in either group (Table 4 and Table 5).

**Table 4:** Subgroup analysis by sex (botulinum group)

Subgroup analysis by sex (botulinum group)	Male (n=11) n(%) or mean±standard deviation	Female (n=40) n(%) or mean±standard deviation
Healing of fissure	7 (64%)	24 (60%)
Time to healing (months)	1.6±1.4	2.1±2.1
Occurrence of incontinence	2 (18%)	9 (23%)
· Feces only	1 (9%)	6 (15%)
· Flatus only	1 (9%)	1 (3%)
· Feces and flatus	0 (0%)	2 (5%)
Requiring botulinum reinjection	2 (18%)	5 (13%)
Requiring further surgery	2 (18%)	8 (20%)

**Table 5:** Subgroup analysis by sex (sphincterotomy group)

Subgroup analysis by sex (sphincterotomy group)	Male (n=29) n(%) or mean±standard deviation	Female (n=11) n(%) or mean±standard deviation
Sphincterotomy group		
Healing of fissure	27 (93%)	9 (82%)
Time to healing (months)	1.5±1.6	1.5±1.3
Occurrence of incontinence	3 (10%)	1 (9%)
· Feces only	1 (3%)	0 (0%)
· Flatus only	0 (0%)	0 (0%)
· Feces and flatus	2 (7%)	1 (9%)
Requiring further surgery	4 (14%)	1 (9%)

## Discussion

Botulinum toxin acts as a muscle relaxant to reduce the release of acetylcholine. The internal anal sphincter is a smooth muscle where acetylcholine inhibits its function. Hence botulinum acts by reducing acetylcholine release in the internal anal sphincter.

Our results demonstrate similar efficacy of botulinum injection for anal fissure to the previous retrospective studies [10-12], although this was inferior in comparison with sphincterotomy. Long-term incontinence was low in both groups. However, there is considerable heterogeneity of botulinum dose and site of administration in the available literature comparing botulinum injection to sphincterotomy.

Previous retrospective evidence has shown a lower recurrence rate of anal fissures following high-dose botulinum toxin injection (80 to 100 units) compared to lower doses (40 vs 47%), without an increase in long-term incontinence [13]. A randomized trial in 2018 showed that injection-related pain and healing rates were similar for both bilateral and unilateral injections, although injection into all four quadrants was not considered in this study [14].

This current study had strengths including the relatively long follow-up of patients (telephone survey at a median of 36 months postoperatively, extending beyond the initial postoperative review) and direct questioning about key complications, such as incontinence, which can be underreported. The limitations of this study include its small size and retrospective nature.

Despite these limitations, our study confirms existing evidence that botulinum toxin injection is an efficacious, less-invasive alternative to sphincterotomy for anal fissures and may be associated with reduced occurrence of persistent incontinence of feces or flatus [9]. Further research and studies are required to go further in-depth into the most effective dosage of botulinum and whether the preparation mode causes a significant change in outcomes.

Current guidelines from the American Society of Colon and Rectal Surgeons endorse lateral internal sphincterotomy as the surgical treatment of choice for anal fissures, although noting the use of botulinum toxin has similar results to topical therapies as a first-line treatment and modest improvement as second-line treatment [15].

Further prospective research is indicated to further investigate the influence of botulinum dose and site of administration on outcomes. This evidence is important to identify the optimal treatment regimen and delineate outcomes compared to lateral internal sphincterotomy to assist in the development of updated consensus recommendations on the use of botulinum toxin for treatment of anal fissures.

## Conclusions

Intrasphincteric injection of botulinum toxin is an effective, less-invasive alternative to sphincterotomy for the surgical treatment of anal fissures, with incontinence usually temporary when it occurs. Further prospective research is needed to compare the dose and location of injection to optimize postoperative outcomes and guide future recommendations.
